# Evolving roles of Data Coordinating Centers in multisite research: Challenges and adaptations from a rapid scoping review

**DOI:** 10.1017/cts.2026.10755

**Published:** 2026-05-15

**Authors:** Yunxi Zhang, Lincy Lal, Soeun Kim, John Michael Swint, David T. Mauger, Aimee Merchlinski, Paula A. Valencia, Beth R. Holmes, Brenda Phillips, Kendall Thomas Baab, Vernon M. Chinchilli

**Affiliations:** 1 Penn State College of Medicine, USA; 2 The University of Texas Health Science Center at Houston, USA; 3 Azusa Pacific University, USA

**Keywords:** Data Coordinating Center, multisite studies, research infrastructure, data quality, regulatory compliance

## Abstract

Data Coordinating Centers (DCCs) play essential roles in multisite clinical and translational research, ensuring consistent protocol implementation, data integrity, and regulatory compliance across geographically and organizationally diverse sites. As study design, regulatory, and technological complexity have evolved, DCC responsibilities have expanded beyond data coordination. This rapid scoping review maps published experiences from academic DCCs to address two questions: (i) What key organizational and operational challenges have been reported? (ii) What solutions and emerging technologies have been adopted in response? We conducted a rapid scoping review in accordance with the PRISMA-ScR (Preferred Reporting Items for Systematic Reviews and Meta-Analyses extension for Scoping Reviews). We searched the peer-reviewed literature (2010–2025) using PubMed, Scopus, and the Web of Science Core Collection. Fifteen reports describing 16 DCCs were included in this review. DCCs faced recurring challenges related to infrastructure development, multisite coordination, regulatory governance, data heterogeneity, and workforce development. Reported adaptations include homegrown modular infrastructure solutions, standardized workflows, streamlined proactive regulatory processes, and workforce investments. By synthesizing evidence on DCC challenges and adaptations, this review provides practical guidance to help DCCs enhance operational feasibility and uphold scientific integrity in multisite clinical and translational research.

## Introduction

Multisite studies are fundamental to clinical and translational research, providing high-quality scientific evidence [1,2]. By leveraging research networks across multiple institutions under a shared protocol, such studies accelerate participant recruitment from diverse populations, thereby enhancing the generalizability of findings and providing the statistical power and precision needed for reliable inference [3]. However, early multisite studies also revealed that achieving consistent study implementation and reliable data across sites required dedicated coordination. In the mid-1960s, when the National Heart Institute initiated a series of large interventional studies, centralized coordination was recognized as essential to ensure data consistency, analytic validity, timely reporting, and effective communication across clinical sites, which later became core responsibilities of Data Coordinating Centers (DCCs) [2,4–8].

In the 1990s, the introduction of the International Conference on Harmonization Good Clinical Practice (ICH-GCP) guidelines and the US Food and Drug Administration (FDA) 21 Code of Federal Regulations (CFR) Part 11 rule on electronic records and signatures substantially expanded the scope of study coordination and the responsibilities of DCCs [9,10]. These international guidelines and national mandates formalized expectations for data quality, documentation, monitoring, and oversight, elevating many coordination efforts that had previously followed scientific best practices to regulatory and compliance requirements [4,11].

In contemporary clinical and translational research, multisite studies are increasingly coordinated through research consortia that span multiple countries, institutions, disciplines, and data environments [12,13]. Correspondingly, the coordination challenges that initially motivated the creation of DCCs have further intensified and diversified. Coordinating across institutions now routinely involves managing heterogeneous data sources and data types, often collected using different systems and standards, which complicates data harmonization and quality assurance processes [12,14,15]. Additionally, DCCs for multisite studies must navigate organizational constraints, including variable institutional policies and contractual requirements, as well as cultural and national boundaries, that affect data access and sharing [12,16,17]. These challenges are further compounded by the need to integrate multidisciplinary expertise across clinical, statistical, and informatics expertise [12,18].

Moreover, the COVID-19 pandemic accelerated the adoption of technological solutions across many multisite studies, including remote informed consent, patient engagement, decentralized data collection, virtual study operations, and artificial intelligence (AI) in diagnostic devices [19]. This further expanded the scope of DCC responsibilities, requiring coordination of technology-enabled study workflows while navigating evolving regulatory requirements in patient recruitment and retention, intervention delivery, and data coordination [20,21]. As a result, these factors have contributed to increasingly complex study workflows, placing substantial organizational and operational demands on DCCs.

Despite the growing recognition of these challenges, the existing literature on DCCs remains fragmented, primarily confined to descriptions of individual studies or specific research programs. Although federal funders, such as the National Institutes of Health (NIH), have issued guidance outlining best practices for DCCs [22,23], these materials largely reflect the expectations and requirements rather than synthesized empirical experience from DCC practices. In particular, existing work has not systematically characterized the organizational and operational challenges DCCs face, nor how DCCs have adapted through changes in technology, governance, workflows, and infrastructure. Given the heterogeneous and evolving nature of the multisite studies DCCs coordinate, this gap warrants a scoping review to consolidate current evidence and experiences. By mapping reported challenges, solutions, and opportunities, this review aims to inform DCC practices that meet current demands and are positioned to adapt to evolving multisite research needs.

### Objective and review question

The objective of this scoping review is to map existing knowledge and experiences on the organizational and operational aspects of DCCs in multisite studies. Specifically, we focus on two interrelated research questions: (i) What key organizational (e.g., governance, resources, workforce) and operational (e.g., data quality, compliance, workflow) challenges have been reported for DCCs? (ii) What solutions, best practices, and emerging technologies have been adopted by DCCs as contemporary adaptations to these challenges?

A preliminary search of PROSPERO, MEDLINE, the Cochrane Database of Systematic Reviews, Open Science Framework, and JBI Evidence Synthesis was conducted, and no current or in-progress scoping reviews or systematic reviews on the topic were identified.

## Materials and methods

This rapid scoping review was conducted and reported in accordance with the PRISMA-ScR guidelines [24], with a protocol registered on the Open Science Framework (osf.io/chtdw) [25].

### Eligibility criteria

Eligibility criteria were defined following the Population, Concept, and Context (PCC) framework. We included publications describing academic DCCs or equivalent entities with a centralized data management function, such as statistical coordinating centers and data management centers. We excluded disease registries, biobanks, and data repositories without active study coordination, as well as DCCs operated by commercial entities, such as contract research organizations.

The review focused on organizational and operational aspects of DCCs, including reported challenges, barriers, lessons learned, governance, compliance, collaboration, data quality, workforce development, and the adoption of emerging technologies. Publications limited to study protocols or methodological innovations without substantive discussion of DCC practice were excluded. Eligible articles described DCCs in multisite settings (with at least two clinical sites) on behalf of research consortia or clinical study networks. Studies limited to single-site, purely local settings or research networks without a specific study were excluded.

Publications reported in English between January 1, 2010, and August 31, 2025, were included. Peer-reviewed journal articles were eligible for inclusion. Editorials, letters, white papers, news briefs, and abstracts without full text were excluded.

### Information sources and selection

Following a pilot test of eligibility criteria, we conducted a systematic search of PubMed (US National Library of Medicine), Scopus (Elsevier), and Web of Science Core Collection (Clarivate Analytics) to identify relevant publications. Full search strategies for each database are listed in the Supplementary Material. Reference lists of included reports were screened and searched manually to identify additional eligible publications. One author (YZ) conducted title and abstract screening, as well as full-text review, to identify potentially eligible records. To ensure the reliability of the screening process, any record whose eligibility was uncertain was elevated for full-text review and discussed with the research team. The screening was managed using Rayyan, a web-based systematic review management platform [26].

### Data charting and synthesis

YZ, LSL, and SK conducted data charting using a data extraction form developed based on the DCC best practices checklist by the National Heart, Lung, and Blood Institute (NHLBI) [23]. YZ extracted data from all included sources, while LSL and SK independently charted subsets of sources, such that each source was charted by two reviewers. Multiple publications on the same DCC were combined into one entry to prevent duplication, with discrepancies resolved through research team discussions.

The data extraction form captured the following information: (1) source metadata (author, year, and journal), (2) study contextual information (country/region of study, research consortium/network, number of clinical sites, and funding source), (3) DCC characteristics (personnel components, organizational setting, and reported responsibilities), (4) challenges, adaptations, and technologies reported for each item outlined in the NHLBI DCC best practices checklist, categorized into four domains: Clinical Study/Trial Operations, Data Management, Quality Assurance/Quality Control, and Human Subjects Protections and Regulatory Affairs [23]. When information was unclear or insufficiently reported, this was documented rather than inferred.

Extracted data were summarized using descriptive tables to identify patterns in DCC characteristics, key challenges, and implemented adaptations across the four domains of NHLBI DCC best practices checklist [23]. To synthesize findings, a narrative synthesis was conducted to organize reported challenges and adaptations into broader organizational and operational categories, facilitating the identification of recurring challenges and potential opportunities.

## Results

### Document selection and characteristics

**Figure 1.**
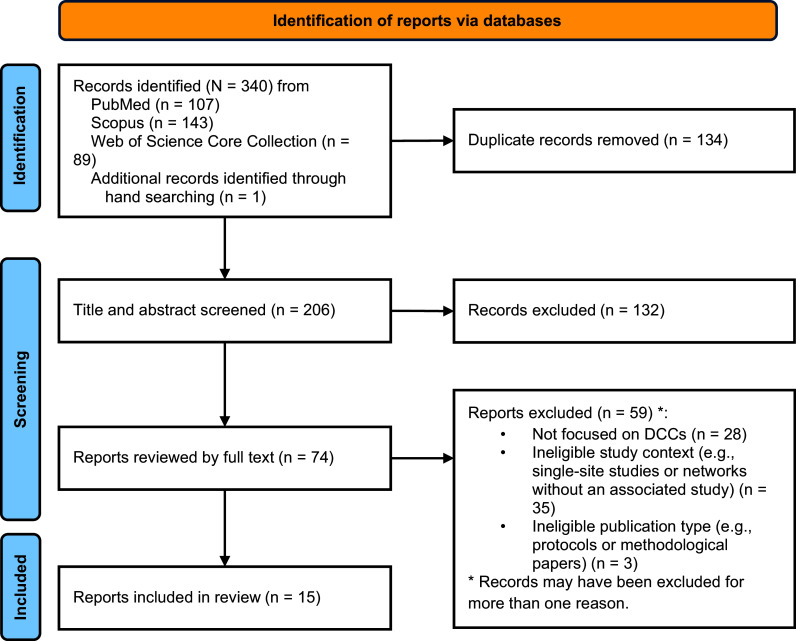
Figure 1 long description. PRISMA flowchart illustrating the search and selection process results for the scoping review.

A total of 340 records were identified from PubMed (*n* = 107), Scopus (*n* = 143), Web of Science Core Collection (*n* = 89), and hand searching (*n* = 1). After deduplication, 206 records remained for title and abstract screening, of which 132 did not meet the inclusion criteria. Following a full-text review of the remaining 74 records, 59 ineligible reports were excluded for the following reasons: (i) not focused on DCCs (*n* = 28), (ii) ineligible study context, such as single-site studies or networks without an associated study (*n* = 35), and (iii) ineligible publication type, including protocols and methodological papers (*n* = 3). This resulted in the inclusion of 15 reports describing experiences from 16 DCCs, two of which described the same DCC [27,28], and one that described three separate DCCs [29]. Figure 1 presents the PRISMA flow diagram for report selection [30].

As summarized in Table 1, these DCCs support multisite clinical research across diverse study portfolios, organizational settings, and funding contexts. The study portfolios were heterogeneous, ranging from late-phase interventional trials, for example, phase III randomized clinical trials (RCTs) [31–33], and large-scale observational studies [34–38], to mixed portfolios encompassing both designs [27–29,39,40]. Geographically, eight DCCs were based in the USA [31,33,35,37–39,41,42], while five coordinated international studies across multiple continents [27,28,32,34,36,40]. Three DCCs described in one report did not specify their study locations [29].

**Table 1.** Characteristics of DCCs described in the included reports Table 1 long description.

Author (year)	Reported study portfolio	Site geographics	Network/consortium: operational period	DCC funding source	DCC organizational setting	Reported DCC scope	
Abebe et al. (2019) [31]	4 phase II–III RCTs: explanatory and pragmatic, parallel and crossover designs	USA	NR	Institutional; Federal (NIH, DoD); Industry (Bayer)	Center for Clinical Trials & Data Coordination (established in 2015), Division of General Internal Medicine, University of Pittsburgh	Providing trial design, conduct, coordination, and analysis expertise; supporting investigator career advancement; Obtaining DCC grants.	
Biswas et al. (2012) [34]	Cross-sectional survey and case–control observational study	International (Africa and Asia)	NR	Foundation (Bill & Melinda Gates)	Cooperative Studies Program Coordinating Center, Department of Veterans Affairs, Perry Point, Maryland	Providing data management support, administrative support, and statistical support.	
Burr et al. (2019) [41]	Network-level multisite studies (individual study designs not specified)	USA	Collaborative Pediatric Critical Care Research Network (CPCCRN): NR*	Federal (NIH)	University of Utah	Coordinating all aspects of network studies, including preparing the CIRB submissions on behalf of the network.	
Cummins et al. (2023) [29]	44 clinical research studies: interventional and observational designs (initiated 2020 – 2022)	Varied geography (individual study sites note specified)	NR	Individual study funding source not specified	3 DCCs at AROs: DCC, University of Utah; Clinical Trial Statistical and Data Management Center, University of Iowa; Data Coordination Unit, Medical University of South Carolina	All three DCCs coordinating data for clinical research, providing research design, clinical data management, study management, study randomization and execution, and analysis, and adoption of decentralization technologies.	
Dyer et al. (2022) [35]	Observational study	USA	Type 1 Diabetes in Acute Pancreatitis Consortium (T1DAPC): NR^†^	Federal (NIH)	Department of Public Health Sciences, Penn State College of Medicine	Providing comprehensive consortium support: project management, administration, financial management, regulatory compliance, scientific coordination, data management, research computing, biostatistics and scientific publications.	
Fulda et al. (2022) [32]	Phase III, double-blind, placebo-controlled RCT	International (Botswana, Brazil, Canada, Haiti, India, Peru, South Africa, Spain, Thailand, Uganda, USA, Zimbabwe)	NR^‡^	Federal (NIH); Industry (Kowa, Gilead, ViiV)	Center for Biostatistics in AIDS Research, Harvard T.H. Chan School of Public Health; Frontier Science; Massachusetts General Hospital Imaging Trials Center	Providing methodological and logistical support for data collection, quality control, and analysis of data; Providing statistical design and analysis; Assisting protocol development, data management; Developing and distributing site performance summary reports internally.	
Gillespie et al. (2021) [36]	Observational study	International (USA, Canada, Europe)	Cure Glomerulonephropathy Network (CureGN): 2013–ongoing (as of 2021)	Federal (NIH)	University of Michigan; Arbor Research Collaborative for Health	Providing data coordination, data query, management, monitoring, and quality control; Conducting site performance monitoring and report; Coordinating biospecimen and pathology repository; Providing coordinator training.	
Huvane et al. (2017) [27] Cross et al. (2023) [28]	Network-level multisite studies, including observational, 510(k), master protocol, phase I–IV IND/IDE clinical trials [28].	International (USA, Colombia, Chile, China, Argentina, Australia, Nicaragua, Saudi Arabia, Lebanon, Singapore, New Zealand, Europe, etc.)	Antibacterial Resistance Leadership Group (ARLG): 2013–ongoing (as of 2024)	Federal (NIH)	The Statistical and Data Management Center was a collaboration among Duke Clinical Research Institute, Frontier Science and Technology Research Foundation, and the Harvard T.H. Chan School of Public Health [27], then led by Biostatistics Center, Department of Biostatistics and Bioinformatics, George Washington University [28].	Ensuring scientific integrity across the study lifecycle (design, conduct, analysis, reporting); Developing and implementing research methods and tools; Educating and mentoring the scientific community [28].	
Krischer et al. (2014) [40]	Pilot studies; Observational studies: case–control, cross-sectional; Surveys; Interventional trials: phase I–III	International	Rare Diseases Clinical Research Network (RDCRN) with 17 consortiaˆ: 2003–ongoing (as of 2014)	Federal (NIH)	Data Management and Coordinating Center, University of South Florida	Providing data management and support; Integrating protocols, forms, and research tools to the research network; Training clinical investigators in rare disease research.	
Major Extremity Trauma Research Consortium (2016) [42]	Observational studies; RCTs	USA	Major Extremity Trauma Research Consortium (METRC): 2009–ongoing (as of 2016)	Federal (NIH, DoD)	DCC, Johns Hopkins Bloomberg School of Public Health	Study design and analytic expertise; data management and quality control; development and sustainability of multicenter research infrastructure; investigator professional development	
Muenzen et al. (2022) [39]	Clinical genomics research projects with heterogeneous study designs and site-specific protocols	USA	Clinical Sequence Evidence-Generating Research (CSER) Consortium: 2017–ongoing (as of 2022)**	Federal (NIH)	University of Washington	Designing, developing, testing, and deploying the infrastructure to aggregate harmonized survey and genomic sequencing data in a secure and centralized way.	
Rahbar et al. (2011) [37]	Observational study	USA	PRospective Observational Multicenter Major Trauma Transfusion (PROMMTT) Consortium: 2008–ongoing (as of 2011)	Federal (NIH)	Center for Clinical and Translational Sciences, University of Texas Health Science Center at Houston	Developing and maintaining study infrastructure; Administering contractual agreements with each clinical site; Standardizing data collection processes and management; Providing training to clinical site personnel; Conducting site monitoring and statistical analysis	
Welch et al. (2017) [38]	Observational study (real-world data integration)	USA	High Value Healthcare Collaborative: NR	Foundation (Laura and John Arnold)	The Dartmouth Institute for Health Policy and Clinical Practice	Centralized data receipt, cleaning, and quality control; iterative data validation and resubmission coordination	
Zhao et al. (2016) [33]	2 phase III RCTs with centralized outcome adjudication	USA	Neurological Emergencies Treatment Trials network: NR; NIH Stroke Trials network: NR	Federal (NIH)	Data Coordination Unit, Medical University of South Carolina	Providing full-scope information support for data management and project management activities; Developing an outcome adjudication infrastructure; regulatory-compliant trial conduct support;	

*Note:* RCT = randomized clinical trial; NIH = National Institute of Health; DCC = Data Coordinating Center; DoD = Department of Defense; CIRB = Centralized Institutional Review Board (IRB); ARO = Academic Research Organization; IND/IDE = Investigational New Drug and Investigational Device Exemption; SC = steering committee;, sIRB = single IRB.

* CIRB development initiated in 2012; full operational period not reported [41].

^†^Recruitment started in 2022; full operational period not reported [35].

^‡^Enrollment started in 2015; full operational period not reported [32].

ˆ17 consortia under the RDCRN: Angelman, Rett and Prader–Willi Syndromes Consortium, Autonomic Rare Diseases Clinical Research Consortium, Brain Vascular Malformation Consortium, Chronic Graft Versus Host Disease Consortium, Dystonia Coalition, Genetic Disorders Mucociliary Clearance Consortium, Inherited Neuropathies Consortium, Lysosomal Disease Network, Nephrotic Syndrome Study Network, North American Mitochondrial Disease Consortium, Porphyrias Consortium, Primary Immune Deficiency Treatment Consortium, Rare Kidney Stone Consortium, Salivary Gland Carcinomas Consortium, Salivary Gland Carcinomas Consortium, Sterol and Isoprenoid Diseases Consortium, Urea Cycle Disorders Consortium, Vasculitis Clinical Research Consortium. Operational period of each consortium not reported [40].

** The CSER phase 2 data coordination and analysis was operated between 2017 and 2023, with its DCC starting in 2019 [39].

Among the 13 DCCs that reported funding sources, most reported experiences from federally funded research, including 11 supported by the NIH [27,28,31–33,35–37,39–42] and two by the Department of Defense (DoD) [31,42]. In addition, two DCCs reported foundation funding [34,38], and two reported industry funding [31,32]. One DCC also reported receiving initial institutional funding to support the infrastructure development [31]. Although all DCCs were situated within academic environments, organizational structures varied. Three DCCs were jointly operated by multiple institutions [27,32,36], while many were embedded within an institutional center that may concurrently coordinate multiple studies or research programs [27–29,31–34,37,40,42].

The reported responsibilities of these DCCs, as summarized in Table 2, extended beyond centralized data management. Although some reports focused on specific DCC functions and may not reflect the full scope of activities, DCC responsibilities consistently spanned the research lifecycle. Data management and quality control was the most commonly reported responsibility (*n* = 14) [27–29,31–40,42], followed by statistical analysis (*n* = 12) [27–29,31,32,34–37,39,40,42], regulatory compliance (*n* = 12) [29,31–37,39–42], and study coordination and administration (*n* = 11) [29,31,33–37,39–42]. Additional responsibilities included study design (*n* = 8) [27–29,31,32,35,37,42], site monitoring (*n* = 8) [27,28,32,35–37,40,42], clinical site personnel training (*n* = 5) [35–37,40,42], investigator career development (*n* = 5) [27,28,31,40,42], scientific publication (*n* = 4) [27,28,35,37], and financial management (*n* = 2) [35,42].

**Table 2.** Frequency of reported DCC responsibilities across included reports Table 2 long description.

DCC responsibility	Number of reports
Data management and quality control	14
Statistical analysis	12
Regulatory compliance	12
Study coordination and administration	11
Study design	8
Site monitoring	8
Clinical site personnel training	5
Investigator career development	5
Scientific publication	4
Financial management	2

*Note*: Some reports focused on specific DCC functions, so the responsibilities shown here may not reflect the full scope of activities performed by the DCC.

DCC = Data Coordinating Center.

### Reported key challenges and adaptations

Table 3 presents a mapping of key DCC challenges and corresponding adaptations implemented across all included publications, aligned with the NHLBI domains and subdomains of the DCC best practices checklist [23]. Challenges were identified across all domains, with the clinical study and trial operations most commonly discussed. This mapping serves as a reference for DCCs encountering similar issues. Table 4 further synthesizes reported challenges into broader organizational and operational categories and distills the reported adaptations into forward-looking opportunities that DCCs may consider for future development and investment.

**Table 3.** Key challenges and adaptations Table 3 long description.

Subdomains	Key challenges	Adaptations implemented
Domain 1: Clinical study/trial operations		
Program initiation for success	Securing funding to develop DCC infrastructure [31].	Leveraging institutional investment and demonstrate its ROI [31].
	Initiating the research program with broad and interdependent operational responsibilities [35,39].	Establishing structured operational units with regular cross-unit communication [35,39].
Communication	Coordinating multidisciplinary teams and high-volume cross-site communication [31,32,34–39].	Dedicated study coordinator as “conduit” between clinical/data/statistics teams [31,32,35,39].
		Implementing structured communication workflows with a standard cadence, using asynchronous communications tools (e.g., project management platforms) with scheduled sync meetings, supported by centralized reporting tools, site performance feedback, and committee oversight [31,32,34–39,42].
Administrative coordination	Complex multi-country logistics and coordination across sites [34].	Centralized coordination supported by standardized procedures, CRFs, and data transfer protocols [34].
	Managing project timelines, resources, and multidisciplinary staff across complex DCC operations [35].	Implementing a matrix management system across core operational units [35].
	Administering funds for the support of consortium-wide research activities [35].	Establishing subaward agreements and a framework for reimbursement [35].
	Efficiency of resource allocation affected by heterogeneity in site enrollment capacity and workload [35,42].	Implementing performance- and workload-based budgeting, with annually negotiated resource allocation [35,42].
Study planning and management	Scientific and operational challenges from heterogeneous study designs, endpoints and diverse data sources [27,28,33,34,37–40].	Developing intricate domain-specific knowledge (e.g., disease, pathogens, interventions, and diagnostics) and promoting effective collaboration with investigators on all study stages, from study design to publication [27,28,37].
		Standardized data collection procedures (CRFs, data dictionaries, and data transfer protocols) and a centralized system [33,34,37–40].
	Regulatory complexity and delays in multi-institutional IRB review for network-level studies [35,37,40–42].	Establishing a structured multi-step sIRB reliance and approval process with a dedicated regulatory liaison [35,41].
		Centralizing IRB oversight through the DCC to streamline regulatory review, with additional site-level support as needed [37,40,41].
	High cost and complexity of integrating decentralized technology into study workflow [29].	Developing site-specific budgets to account for technology costs, and conducting of cost–benefit analysis to guide decentralized infrastructure investment [29].
	Integrating data from decentralized technologies (e.g., ePROs and wearables) into centralized data collection platforms [29].	Platform-level integration planning with testing across devices, environments, and automated workflows [29].
	Maintaining high retention rates in a long-term, global trial [32].	Developing and implementing data-driven standardized site performance metrics, regular evaluations, structured communication with feedback and targeted support [32].
	Sustaining investigator career development [31,40].	Supporting grant development for network investigators [31].
		Integrating investigator training with scholarly activities [40].
	Patient recruitment challenges [40,42].	Developing and utilizing a web-based Patient Contact Registry for recruitment across broad geographies where traditional site-based recruitment is inefficient [40].
		Establishing a large-scale multicenter research network and a startup registry to support feasibility assessment and enrollment monitoring [42].
Publication and presentation development	Managing publication practices and authorship attribution in large multisite studies [27,28,31,35,37,42].	Defining network publication policies and leading or supporting clinical, methodological, and educational manuscript development [27,28,31,35,37,42].
Domain 2: Data management		
Global and data security	Limited suitability of DM platforms for secure, multi-country data collection and transfer [34,39].	Standardized data collection procedures (CRFs and data transfer protocols) and a centralized system [34,39].
	Cybersecurity and integration risks from multi-vendor digital health environments and decentralized data sources [29].	Risk assessments, platform integration planning, and ongoing configuration, testing, and support to ensure data integrity and interoperability [29].
Data systems	High cost and lack of customization and integrative features in third-party CTMS [31,33].	Developing and employing a homegrown, regulatory-compliant, modular CTMS/DMS to support these functions [31,33,35].
	Supporting regulatory tracking and operational coordination [35].	
	Managing and validating PRO data collection and integration [35].	
	Language heterogeneity of CRFs across participating countries [34].	Standardized multilingual CRFs with centralized translation and validation [34].
	Handling diverse data types, including biospecimen samples and imaging data (CT/MRI scans) [32,35,37].	Implementing secure workflows for de-identified biospecimen tracking and anonymized imaging data transfer, with validation and reconciliation [32,35,37].
Randomization	Ineligible randomization due to data entry errors [31].	Using a dynamically updated web form that pulled data from multiple forms to confirm eligibility before randomization [31].
Reporting	Ensuring high-quality, timely, and complete data across multisite studies during ongoing accrual [32,33,35–39,42].	Using standardized data workflows with real-time validation, and automation, complemented by centralized, automated reporting of data quality and site performance metrics [32,33,35–39,42].
Analysis	Designing appropriate statistical models [27–29,35,37].	Designing and implementing appropriate statistical methodologies to ensure scientific integrity across the study lifecycle [27–29,35,37].
Domain 3: Quality control/quality assurance		
Quality assurance	Decentralized research technology requires evolving competencies [29].	Developing targeted training programs, education initiatives, and methodological research to support quality and good practices [29].
	Ensuring consistent protocol implementation and data quality across sites [35,36,42].	Establishing a role-based user training, registration and certification system tracked in DMS [35,42].
		Implementing standardized onboarding and refresher training [36].
	Limited understanding of clinical trial fundamentals within the research community, affecting scientific rigor and clinical utility [27,28].	Developing and disseminating educational resources and scholarly publications on clinical trial design and statistical methodologies [27,28].
	Ensuring oversight of data quality, site monitoring, participant safety, and study progress [33,35–37,39,42].	Integrating accrual, data quality, and safety data with QA monitoring and reporting workflows [33,35–37,39,42].
Quality control	Costly and inefficient on-site monitoring [31].	Employing a risk-based central monitoring model focusing on critical data points per FDA guidance [31].
	Participant non-responsiveness and technical issues affecting data quality in decentralized research [29].	Prospective monitoring of data accuracy and transmissions with statistical oversight and participant education [29].
Domain 4: Human subjects protection/regulatory affairs		
Human subject protections	Consenting participants in decentralized and FDA-regulated trials [29].	Implementing eConsent and e-signature workflows [29].
	Delays from sequential, multi-level human regulatory review process [42].	Implementing benchmark tracking and active site monitoring to reduce approval timelines [42].
	Harmonizing consent language across sites in the absence of a CIRB/sIRB [39,42].	Aligning network-level data governance to support consistent consent language and data use across sites, guided by a multidisciplinary data access group [39].
FDA related regulatory requirements	Meeting 21 CFR Part 11 compliance for electronic records/signatures [31].	Developing and employing a homegrown, regulatory-compliant CTMS [31].
OHRP related regulatory	Managing regulatory document lifecycles [31].	Developing and employing a homegrown CTMS with eMRF to track and alert on document expirations [31].

ROI = return-on-investment; CIRB = Centralized Institutional Review Board (IRB); CRF = case report form; CTMS = clinical trial management system; eMRF = electronic master regulatory files; HRPP = Human Research Protection Program; DCC = Data Coordinating Center; DMS = data management system; ePRO = electronic patient-reported outcomes; OHRP = Office for Human Research Protection; CFR = Code of Federal Regulations.

**Table 4.** Cross-domain synthesis of organizational and operational challenges and potential opportunities Table 4 long description.

Challenge category	Representative challenges	Potential opportunities
Organizational		
Establishing and sustaining DCC infrastructure	Securing funding for infrastructure development [29,31]. High cost and lack of customization features in third-party CTMS/DMS for diverse study needs [31,33,34,39].	Proactively demonstrate value to secure institutional and extramural funding aligning network goals with consideration of cost efficiency, for example, ROI, cost–benefit analysis [29,31]. Develop and adopt regulatory-compliant, modular, homegrown systems, offering a scalable alternative to costly third-party solutions [31,33,35].
Resource allocation and site heterogeneity	Aligning resources with heterogeneous site enrollment capacity and workload [32,35,42].	Implement workload- and performance-based budgeting models [35,42].
Workforce development and collaboration	Building domain-specific knowledge and clinical trial fundamentals [27,28]. Sustaining long-term investigator engagement and career development [27–29,31,35,40]. Adapting to evolving staff competencies required for variable study designs, operational contexts, and regulatory requirements [31,32,35–37,41,42].	Embed professional development into DCC missions, supporting grant writing and scholarly output for network investigators to foster long-term engagement and scientific growth [27–29,31,35,40]. Integrate continuous, role-based training into DCC operations, educating both internal staff and the wider network [31,32,35–37,41,42].
Regulatory governance and compliance	Navigating regulatory complexity and delays in multi-institutional IRB review processes [35,37,40–42]. Meeting evolving regulatory requirements [29,31,33]. Harmonizing consent language and governance across sites [37,39].	Monitor regulatory timelines and establish benchmark-based oversight [37,42]. Implement CIRB/sIRB with the DCC acting as the regulatory liaison [35,41,42]. Align network-level data governance to support consistent consent language and data use across sites [37,39].
Operational		
Multisite coordination and communication	Coordinating multidisciplinary teams and high-volume cross-site communication [31,32,34–39]. Ensuring oversight of study progress, safety, and data quality across all sites [33,35–37,39,42]. Managing publication practices and authorship attribution in large multisite studies [27,28,31,35,37,42].	Implement structured communication workflows with standard cadence, using asynchronous and scheduled sync approaches [31,32,34–39,42]. Centralize oversight through integrated dashboards that provide real-time, automated metrics on enrollment, data quality, and site performance [32,33,35–39,42]. Establish and enforce clear, predefined network publication policies and authorship guidelines to manage expectations and credit sharing [27,28,31,35,37,42].
Study design and data management	Addressing scientific and operational challenges from heterogeneous study designs, endpoints and diverse data sources [27,28,33,34,37–40].	Embed DCC involvement early in study planning and standardized data collection procedures (CRFs, data dictionaries, and data transfer protocols) and a centralized system [27,28,33,34,37–40].
Data integrity and site monitoring	Ensuring high-quality, timely, and complete data across multisite studies during ongoing accrual [31–39,42]. Managing cybersecurity and integration risks from multi-vendor digital health environments [29].	Integrate accrual, data quality, and safety data into centralized monitoring workflows [31–39,42]. Conduct risk assessments, platform integration planning, and ongoing configuration, testing, and support to ensure data integrity and interoperability [29].

CIRB = Centralized Institutional Review Board (IRB); CTMS = clinical trial management system; CRF = case report form; DCC = Data Coordinating Center; DMS = data management system; ROI = return-on-investment; sIRB = single IRB.

### Organizational challenges and adaptations

At the organizational level, establishing and sustaining DCC infrastructure is a fundamental challenge, primarily due to the need to secure stable funding for infrastructure development and to the limited customization of third-party clinical trial management systems (CTMS) or data management systems (DMS) to meet diverse and evolving study needs [29,31,33,34,39]. Studies highlighted the importance of demonstrating institutional and network-level value through cost efficiency considerations to support long-term sustainability [29,31]. For example, the DCC at the University of Pittsburgh described intramural funding as supporting initial infrastructure development, with the expectation that the DCC would become self-sustaining through recurring projects over time [31]. As study needs evolve, DCCs often require continuous infrastructure enhancements, which may be constrained by limited budgets, underscoring the need for formal cost–benefit analyses to guide investment decisions [29]. Additionally, given the high cost and limited flexibility of commercial CTMS and DMS platforms [31,33,34,39], many DCCs reported developing and implementing regulatory-compliant, modular systems to support heterogeneous study portfolios [31,33,35]. The homegrown CTMS developed by the DCC at the University of Pittsburgh integrates multiple functional components, including electronic data capture, eligibility and randomization, drug and external data tracking, safety reporting, data and safety monitoring, statistical analysis and reporting, data sharing, and regulatory compliance [31]. Zhao and Pauls [33] described an outcome adjudication module within their homegrown CTMS. The DCC of the Type 1 Diabetes in Acute Pancreatitis Consortium (T1DAPC) also reported multiple core modules to meet study needs, including participant data and survey management, error tracking, document management, reimbursement tracking, sample tracking, and image data transmission [35].

Resource allocation is challenged by substantial heterogeneity in site environments, capacity, and workload. In coordinating a global trial, Fulda et al. highlighted geographic variation in personnel costs affecting site staffing and performance [32]. In response, DCCs may consider implementing performance- and workload-based budgeting models. For example, the Major Extremity Trauma Research Consortium (METRC) negotiated budgets annually based on site performance and projected workload for the upcoming year [42], while the DMS used by the T1DAPC DCC overlays the visit structure defined in the study protocol with reimbursement rules to adjust payment and set grant limits, aligning payment with actual study activity and avoiding overpayment [35].

Workforce development represents another critical area for DCCs, encompassing both investigators and staff. For investigators, DCCs face challenges in building domain-specific knowledge and clinical trial fundamentals, as well as sustaining long-term investigator engagement and career development [27–29,31,35,40]. For staff members, DCCs must adapt to evolving competency requirements driven by diverse study designs, operational contexts, and regulatory requirements [31,32,35–37,41,42]. Across included studies, reported adaptations pointed to opportunities to embed professional development into DCC missions, supporting grant writing and scholarly output for network investigators to foster long-term engagement and scientific growth [27–29,31,35,40]. For example, the Antibacterial Resistance Leadership Group (ARLG) DCC emphasized developing statistical methodology and disseminating educational resources to the scientific community [27,28]. For staff members, DCCs may consider integrating continuous, role-based training and certification into their operations to ensure competency and protocol adherence across sites [31,32,35–37,41,42]. The T1DAPC DCC embedded training and certification within its DMS to ensure that study activities were performed only by certified staff [35].

DCCs also face regulatory and governance challenges, particularly in multi-institutional and multinational IRB processes, evolving regulatory requirements, and heterogeneity in consent language and governance across sites [29,31,33,35,37,39–42]. Reports highlighted adaptations such as benchmark-based monitoring of regulatory approval timelines [37,42], the use of centralized IRB (CIRB) or single IRB (sIRB) models with the DCC acting as a regulatory liaison [35,41,42], and the development of harmonized, network-level data governance frameworks to promote consistency in consent language and data use across sites [37,39].

### Operational challenges and adaptations

At the operational level, DCCs commonly reported challenges related to multisite coordination and communication, such as managing high-volume cross-site communication [31,32,34–39], oversight of study progress, safety, and data quality [33,35–37,39,42], and managing publication practices and authorship attribution across research networks [27,28,31,35,37,42]. Across studies, implemented adaptations suggest opportunities to formalize communication workflows with standardized cadence, integrate asynchronous tools (e.g., email and project management platforms such as Basecamp) with scheduled synchronization to support consensus-building across sites, and use integrated dashboards to centralize real-time oversight of enrollment, data quality, and site performance [31–39,42]. The emphasis on experienced study coordinators as central points of contact further indicates the importance and challenge of coordination roles in bridging multidisciplinary teams and heterogeneous site environments [31,32,35,39]. The matrix management system adopted by the T1DAPC DCC facilitates cross-unit communication within the DCC and project timeline management [35]. Additionally, the commonly adopted predefined publication policies and authorship guidelines underscore the importance of proactively managing the expectations and accountability for scholarly outputs [27,28,31,35,37,42].

Operational challenges related to study design and data management are primarily driven by heterogeneity in study designs, endpoints, and data sources across multisite research networks, creating both scientific and operational complexity for DCCs [27,28,33,34,37–40]. These challenges underscore opportunities for early and continuous DCC engagement to ensure methodological alignment and data harmonization. Specifically, reported adaptations highlight the use of centralized systems and standardized data collection tools, such as the development of case report forms (CRFs), data dictionaries, and data transfer protocols [27,28,33,34,37–40]. Muenzen et al. further highlighted the importance of planning and budgeting for data coordination activities as early as possible to minimize downstream complications [39]. When CRFs cannot be implemented, for example, data collection through electronic health records (EHRs), data specification documents were developed to define key elements, including case definitions, to support data consistency across sites [38].

Moreover, ensuring protocol adherence requires high-quality, timely, and complete data during ongoing accrual, which poses another persistent operational challenge [32,33,35–39,42]. To address this, DCCs commonly develop centralized monitoring workflows that integrate accrual metrics, data quality indicators, and safety information [31,32,34–39,42]. Such monitoring approaches are often implemented alongside adaptations for multisite coordination and communication [31,32,34–39,42]. Furthermore, the adoption of decentralized technologies introduces distinct cybersecurity and data integration risks from multi-vendor digital health environments, posing data security challenges [29]. Mitigating these risks requires proactive technology governance, including risk assessments, platform integration planning, and ongoing configuration, testing, and technical support to ensure data integrity and interoperability from the local device to the central data infrastructure [29].

## Discussion

In this scoping review, we synthesized published experiences from academic DCCs supporting multisite research and identified recurring organizational and operational challenges, corresponding adaptations, and emerging opportunities. In addition to their traditional coordination functions, many DCCs play leadership roles within consortia, particularly in facilitating data sharing, dissemination of results, and broader outreach activities. Accordingly, our findings highlight the expanding scope of DCC responsibilities and underscore the need for adaptive coordination that integrates governance, technology, and workforce capacity to support contemporary multisite clinical and translational studies.

At its core, the primary role of DCCs is to function as the central hub for communication and coordination across geographically dispersed studies. Effective coordination is foundational to consistent protocol implementation and adherence. Across the reviewed studies, this coordinating function has been increasingly supported by standardized workflows, centralized data platforms, and shared monitoring systems that collectively facilitate communication, data management, and site oversight. Notably, substantial heterogeneity in study designs, site capabilities, data sources, and regulatory environments was consistently reported, precluding a one-size-fits-all model for DCCs. In response, many DCCs have developed and adopted modular or scalable approaches that integrate components and leverage existing tools, rather than relying on a single comprehensive platform [31,33–35]. Such strategies allow DCCs to tailor infrastructure to meet study-specific needs while maintaining reproducible operational processes. Beyond the included literature, a recent report described a cost-effective, regulatory-compliant safety case management workflow that replaced specialized pharmacovigilance software that was incompatible with other clinical research software and required an annual license fee, thereby illustrating the value of workflow redesign to improve interoperability, efficiency, and effectiveness, while maintaining compliance [43].

Beyond coordination, regulatory governance represents a critical responsibility of DCCs. Navigating multi-institutional ethics review processes, meeting evolving regulatory requirements and standards (e.g., 21 CFR Parts 11 and 312, and ICH E6), and harmonizing consent language and data use across sites were commonly reported challenges [29,31,33,35,37,39–42]. Differences in funder requirements, state and institutional policies, and interoperability constraints across technology platforms at participating sites can make each DCC’s human-subjects protection landscape unique. In response, DCCs have spearheaded efforts to streamline compliance through developing CIRB or sIRB processes with the DCC as the regulatory liaison [35,41,42], benchmarking regulatory timelines [37,42], and aligning network-level data governance [37,39]. These governance structures not only supported regulatory compliance but also facilitated a more efficient study startup.

Technological advances present both opportunities and demands in conducting multisite studies. Technology-enabled workflows offer greater flexibility in coordination, communication, and data management [29,31–34,37,39,40,42], and decentralized approaches, such as telemedicine, wearable devices, electronic patient-reported outcomes (ePROs), online recruitment portals, and eConsent, have extended engagement with populations that were previously hard to reach [29,40]. For example, telemedicine allows health services to be delivered to participants at remote locations without transportation, while wearable devices facilitate real-time patient monitoring and data collection [19,29,44]. However, these advances also introduce new operational demands, including increased requirements for data validation, system integration, cybersecurity, and regulatory oversight. For example, ePROs allow participants to report outcome data directly through web- or mobile-based applications, introducing additional requirements for data validation and quality control, while eConsent, which digitizes the informed consent process, must meet regulatory and compliance standards [19,29]. These challenges underscore the need for DCCs to adapt their infrastructure, governance, and operational capacity to keep pace with evolving study needs. In this context, careful consideration of efficiency and cost efficiency becomes increasingly important when academic DCCs invest in and upgrade underlying infrastructure to support technology-enabled study workflows.

Ultimately, the ability of DCCs to ensure scientific integrity in large multisite clinical studies also depends on the strength and integration of their multidisciplinary workforce and the broader scientific community they engage with. As modern studies become more complex, expanding regulatory requirements and rapidly evolving data systems, combined with the enduring importance of methodological rigor, place increasing demands on workforce competence and statistical and methodological expertise [27,28,45,46]. Our review highlighted the critical role of experienced clinical research professionals in facilitating effective communication among multidisciplinary teams and across multiple sites [31,32,35,39]. It also underscored the importance of ongoing, role-based training and support for DCC internal staff and the wider research network [31,32,35–37,41,42]. These findings align with prior work emphasizing the need to strengthen competencies among clinical research professionals who support clinical studies, including the development of competency-based frameworks, educational curricula, and mentorship programs [47–50]. Meanwhile, ensuring scientific rigor among DCC-supported research requires continued development and dissemination of statistical and methodological expertise, particularly as study designs, informatics, and analytic expectations evolve [27,28]. Moreover, several reviewed DCCs emphasized the importance of engaging network investigators in manuscript and grant development [27–29,31,35,40]. This involvement not only promotes investigator career growth and ongoing engagement within the network but also reinforces the continuity of network research by creating a pipeline of investigators who continue to advance and expand the scientific agenda. Together, investments in workforce competence and methodological capacity enable DCCs to adapt to evolving research environments while maintaining scientific quality and supporting the continuity of network research.

Looking ahead, the role of DCCs is likely to further evolve with the rapid advancement of emerging technologies, including AI, robotics, and automation [20,51]. These technologies are increasingly being integrated into biospecimen processing, medical devices, decentralized trials, and broader study infrastructure [20,21,52], contributing to growing complexity and heterogeneity in multisite research. In this context, DCCs will need to remain adaptive, developing robust workflows and infrastructure while meeting the regulatory requirements. DCC and clinical site workforce development will be essential to support the effective integration of emerging technologies in study implementation and DCC workflows. Moreover, academic DCCs are well positioned to contribute to the development of best practices and to work closely with investigators to translate emerging scientific and technological advances into practical research evidence. Collaborative initiatives such as the UNIversity data COoRdinating ceNters (UNICORN) Network provide an example of how academic DCCs can align efforts to support innovation, standardization, and knowledge sharing across institutions [53].

## Limitations

This scoping review has several limitations. First, as a rapid scoping review, this study prioritized breadth and timeliness over exhaustive coverage. Although multiple databases were searched and reference lists were screened, relevant reports, particularly unpublished experiences, internal reports, or gray literature describing DCC practices, were not captured. Second, the included literature was heterogeneous in study context, scope, and level of detail, and all reports were descriptive. For example, reported DCC responsibilities varied depending on research network structures, particularly when clinical coordination was managed by entities separate from the DCC [27,28]. Third, our synthesis relied on reported experiences. Although the NHLBI DCC best practices checklist was used to guide data collection [23], the extracted data reflect the focus and scope of published reports and may not fully capture the complexity of day-to-day challenges or the implementation of adaptations in practice. This may introduce publication bias, whereby certain operational challenges are underrepresented. For example, cross-institutional email distribution may be unreliable due to evolving institutional email security policies, requiring an alternative communication infrastructure to ensure consistent coordination among teams. Such challenges are commonly encountered in the multi-institutional coordination but not described in the reviewed reports.

## Conclusions

Multisite clinical studies emerged to generate robust evidence but quickly revealed the need for dedicated coordination, giving rise to DCCs as the “hub around which various clinical centers must revolve.” [2,4–8,54,55] As regulatory, technological, and methodological complexity have evolved, DCC responsibilities have expanded beyond centralized data management to encompass comprehensive study coordination, governance, and scientific support. In response to this evolution, this rapid scoping review synthesizes published experiences to characterize how academic DCCs have adapted. Our findings underscore the importance of adapting to study heterogeneity through modular infrastructure solutions, centralized and standardized workflows, proactive governance, and workforce investment, which is critical to operational feasibility and scientific integrity of contemporary multisite research.

## Supporting information

10.1017/cts.2026.10755.sm001 Zhang et al. supplementary material Zhang et al. supplementary material

## Supplementary material

The supplementary material for this article can be found at https://doi.org/10.1017/cts.2026.10755.

## Figure 1. Long description

The flowchart begins with the identification phase, where 340 records are identified from various databases: 107 from PubMed, 143 from Scopus, 89 from Web of Science Core Collection, and 1 additional record through hand searching. Duplicate records, totaling 134, are removed. The next phase involves screening titles and abstracts of 206 records, excluding 132 records. Reports are then reviewed by full text, with 74 reports assessed. In the final phase, 59 reports are excluded for various reasons: 28 not focused on Data Coordinating Centers, 35 with ineligible study context, and 3 with ineligible publication type. Ultimately, 15 reports are included in the review.

Navigate back to Figure 1..

## Table 1. Long description

A table comparing characteristics of Data Coordinating Centers (DCCs) in clinical research. The table has eleven rows and eight columns, detailing reported study design, site geography, network consortium, funding source, DCC organizational setting, DCC organizational structure, reported DCC scope, and references. Each row provides specific details for different DCCs, including study types such as phase III randomized clinical trials and large-scale observational studies, geographical locations ranging from the USA to international settings, and various funding sources. The table highlights the diversity in study portfolios, organizational settings, and funding contexts supported by these DCCs.

Navigate back to Table 1..

## Table 2. Long description

The table presents the frequency of reported DCC responsibilities across included reports. It consists of two columns and eleven rows. The first column lists the DCC responsibilities, and the second column shows the number of reports for each responsibility. The responsibilities include data management and quality control with fourteen reports, statistical analysis with twelve reports, regulatory compliance with twelve reports, study coordination and administration with eleven reports, study design with eight reports, site monitoring with eight reports, clinical site personnel training with five reports, investigator career development with five reports, scientific publication with four reports, and financial management with two reports.

Navigate back to Table 2..

## Table 3. Long description

A table mapping key challenges and corresponding adaptations implemented across various publications, aligned with the NHLBI domains and subdomains of the DCC best practices checklist. The table highlights challenges identified in all domains, with a particular focus on clinical study and trial operations. It serves as a reference for Data Coordinating Centers encountering similar issues, and synthesizes reported challenges into broader organizational and operational categories, offering forward-looking opportunities for future development and investment.

Navigate back to Table 3..

## Table 4. Long description

The table presents a detailed comparison of organizational and operational challenges and potential opportunities in DCC infrastructure. It includes categories such as establishing and sustaining DCC infrastructure, resource allocation, workforce development, regulatory governance, multisite coordination, study design, and data integrity. Each category lists representative challenges and potential opportunities. The table has multiple rows and columns, with headers indicating the challenge category, representative challenges, and potential opportunities. Notable trends include the need for securing funding, adapting to regulatory requirements, and implementing structured communication workflows.

Navigate back to Table 4..

## References

[1] National Center for Advancing Translational Sciences. NCATS strategic plan for 2025-2030, secondary NCATS strategic plan for 2025-2030, (https://ncats.nih.gov/sites/default/files/2025-07/NCATS-2025-Strategic-Plan-508.pdf) Accessed February 2, 2026.

[2] Greenberg BG. Organization, review, and administration of cooperative studies (Greenberg report): a report from the Heart Special Project Committee to the National Advisory Heart Council, May 1967. Control Clin Trials 1988;9:137–148. doi: 10.1016/0197-2456(88)90034-7. 3396364

[3] Unverzagt S , Prondzinsky R , Peinemann F. Single-center trials tend to provide larger treatment effects than multicenter trials: a systematic review. J Clin Epidemiol. 2013;66:1271–1280. doi: 10.1016/j.jclinepi.2013.05.016. 23972520

[4] Cook TD , DeMets DL. Introduction to statistical methods for clinical trials. Boca Raton, FL: Chapman and Hall/CRC Press Taylor & Francis Group, 2007.

[5] Meinert CL , Heinz EC , Forman SA. Role and methods of the coordinating center. Control Clin Trials. 1983;4:355–375. doi: 10.1016/0197-2456(83)90022-3. 6675890

[6] Coordinating Center Models Project Research Group. Coordinating Center Models Project: a study of coordinating centers in multicenter clinical trials IV. Terminology. Bethesda: National Heart. Bethesda, MD: Division of Heart and Vascular Diseases, National Heart, Lung, and Blood Institute, 1979.

[7] Meinert CL. Organization of multicenter clinical trials. Control Clin Trials 1981;1:305–312. doi: 10.1016/0197-2456(81)90033-7. 7261620

[8] DuChene AG , Hultgren DH , Neaton JD , et al. Forms control and error detection procedures used at the coordinating center of the multiple risk factor intervention trial (MRFIT). Control Clin Trials 1986;7:34s–45s. doi: 10.1016/0197-2456(86)90158-3. 3802845

[9] International Conference on Harmonisation Working Group. ICH harmonised tripartite guideline: Guideline for good clinical practice E6 (R1) 1996, secondary ICH harmonised tripartite guideline: Guideline for good clinical practice E6 (R1) 1996, (https://www.ywpjlm.com/Uploads/202501/6788cdb7cf631.pdf) Accessed February 2, 2026.

[10] U.S. Food and Drug Administration. Electronic records; electronic signatures (21 CFR part 11), secondary electronic records; electronic signatures (21 CFR part 11), (https://www.govinfo.gov/content/pkg/FR-1997-03-20/pdf/97-6833.pdf) Accessed January 5, 2026.

[11] Gallin JI , Ognibene FP , Johnson LL. Principles and practice of clinical research. Cambridge, MA: Academic Press, 2017.

[12] Austin CP. Opportunities and challenges in translational science. Clin Transl Sci. 2021;14:1629–1647. doi: 10.1111/cts.13055. 33982407 PMC8504824

[13] Goodlett D , Hung A , Feriozzi A , et al. Site engagement for multi-site clinical trials. Contemp Clin Trials Commun. 2020;19:100608. doi: 10.1016/j.conctc.2020.100608. 32685765 PMC7358177

[14] Castano S , De Antonellis V. Global viewing of heterogeneous data sources. IEEE Trans Knowl Data Eng. 2001;13:277–297. doi: 10.1109/69.917566.

[15] Le Sueur H , Bruce IN , Geifman N. The challenges in data integration - heterogeneity and complexity in clinical trials and patient registries of systemic lupus erythematosus. BMC Med Res Methodol. 2020;20:164. doi: 10.1186/s12874-020-01057-0. 32580708 PMC7313210

[16] Gudi N , Kamath P , Chakraborty T , et al. Regulatory frameworks for clinical trial data sharing. Scoping Review J Med Internet Res. 2022;24:e33591. doi: 10.2196/33591. 35507397 PMC9118011

[17] Lidz CW , Pivovarova E , Appelbaum P , et al. Reliance agreements and single IRB review of multisite research: concerns of IRB members and staff. AJOB Empir Bioeth. 2018;9:164–172. doi: 10.1080/23294515.2018.1510437. 30285561 PMC6309766

[18] Ohmann C , Banzi R , Canham S , et al. Sharing and reuse of individual participant data from clinical trials: principles and recommendations. BMJ Open 2017;7:e018647. doi: 10.1136/bmjopen-2017-018647. PMC5736032 29247106

[19] Cummins MR , Soni H , Ivanova J , et al. Narrative review of telemedicine applications in decentralized research. J Clin Transl Sci. 2024;8:e30. doi: 10.1017/cts.2024.3. 38384915 PMC10880018

[20] Rosa C , Marsch LA , Winstanley EL , et al. Using digital technologies in clinical trials: current and future applications. Contemp Clin Trials 2021;100:106219. doi: 10.1016/j.cct.2020.106219. 33212293 PMC8734581

[21] Dorsey ER , Kluger B , Lipset CH. The new normal in clinical trials: decentralized studies. Ann Neurol. 2020;88:863–866. doi: 10.1002/ana.25892. 32869367

[22] National Institute of Diabetes and Digestive and Kidney. Guidance for data coordinating centers (DCCs) management of NIDDK clinical cooperative agreements, secondary guidance for data coordinating centers (DCCs) management of NIDDK clinical cooperative agreements. (https://www.niddk.nih.gov/-/media/Files/Research-Funding/Process/NIDDK-Guidance_DCC-Management-of-CCAs_Final_Version-1,-d-,1_OD-Approved_External-Website.pdf) Accessed January 1, 2026.

[23] National Heart, Lung, and Blood Institute. Compendium of best practices for data coordinating centers, secondary compendium of best practices for data coordinating centers, (https://www.nhlbi.nih.gov/events/2011/compendium-best-practices-data-coordinating-centers) Accessed January 1, 2026.

[24] Tricco AC , Lillie E , Zarin W , et al. PRISMA extension for scoping reviews (PRISMA-ScR): checklist and explanation. Ann Intern Med. 2018;169:467–473. doi: 10.7326/M18-0850. 30178033

[25] Zhang Y , Lal LS , Kim S , et al. Challenges and opportunities of data coordinating centers: a rapid scoping review protocol. (https://osf.io/chtdw/) Accessed January 2, 2026.

[26] Ouzzani M , Hammady H , Fedorowicz Z , et al. Rayyan—a web and mobile app for systematic reviews. Syst Rev. 2016;5:210. doi: 10.1186/s13643-016-0384-4. 27919275 PMC5139140

[27] Huvane J , Komarow L , Hill C , et al. Fundamentals and catalytic innovation: the statistical and data management center of the antibacterial resistance leadership group. Clin Infect Dis. 2017;64:S18–23. doi: 10.1093/cid/ciw827. 28350899 PMC5848245

[28] Cross HR , Greenwood-Quaintance KE , Souli M , et al. Under the hood: the scientific leadership, clinical operations, statistical and data management, and laboratory centers of the antibacterial resistance leadership group. Clin Infect Dis. 2023;77:S288–294. doi: 10.1093/cid/ciad529. 37843120 PMC10578052

[29] Cummins MR , Burr J , Young L , et al. Decentralized research technology use in multicenter clinical research studies based at U.S. academic research centers. J Clin Transl Sci. 2023;7:e250. doi: 10.1017/cts.2023.678. 38229901 PMC10790101

[30] Page MJ , McKenzie JE , Bossuyt PM , et al. The PRISMA 2020 statement: an updated guideline for reporting systematic reviews. BMJ. 2020;2021:372. doi: 10.1136/bmj.n71. PMC8005924 33782057

[31] Abebe KZ , Althouse AD , Comer D , et al. Creating an academic research organization to efficiently design, conduct, coordinate, and analyze clinical trials: the center for clinical trials & data coordination. Contemp Clin Trials Commun. 2019;16:100488. doi: 10.1016/j.conctc.2019.100488. 31763494 PMC6861639

[32] Fulda ES , Fichtenbaum CJ , Kileel EM , et al. The importance of methods for site performance evaluation in REPRIEVE, a longitudinal, global, multicenter trial. Contemp Clin Trials 2023;124:107035. doi: 10.1016/j.cct.2022.107035. 36462699 PMC9891172

[33] Zhao W , Pauls K. Architecture design of a generic centralized adjudication module integrated in a web-based clinical trial management system. Clin Trials 2016;13:223–233. doi: 10.1177/1740774515611889. 26464429 PMC4785064

[34] Biswas K , Carty C , Horney R , et al. Data management and other logistical challenges for the GEMS: the data coordinating center perspective. Clin Infect Dis. 2012;55:S254–261. doi: 10.1093/cid/cis755. 23169938 PMC3502309

[35] Dyer AM , Baab KT , Merchlinski A , et al. Rationale and development of a data coordinating center to support the type 1 diabetes in acute pancreatitis consortium. Pancreas 2022;51:604–607. doi: 10.1097/mpa.0000000000002075. 36206466 PMC9555872

[36] Gillespie BW , Laurin L-P , Zinsser D , et al. Improving data quality in observational research studies: report of the cure glomerulonephropathy (CureGN) network. Contemp Clin Trials Commun. 2021;22:100749. doi: 10.1016/j.conctc.2021.100749. 33851061 PMC8039553

[37] Rahbar MH , Fox EE , del Junco DJ , et al. Coordination and management of multicenter clinical studies in trauma: experience from the PRospective observational multicenter major trauma transfusion (PROMMTT) study. Resuscitation 2012;83:459–464. doi: 10.1016/j.resuscitation.2011.09.019. 22001613 PMC3303947

[38] Welch G , von Recklinghausen F , Taenzer A , et al. Data cleaning in the evaluation of a multi-site intervention project. EGEMS (Wash DC). 2017;5:4. doi: 10.5334/egems.196. PMC5983076 29881755

[39] Muenzen KD , Amendola LM , Kauffman TL , et al. Lessons learned and recommendations for data coordination in collaborative research: the CSER consortium experience. HGG Adv. 2022;3:100120. doi: 10.1016/j.xhgg.2022.100120. 35707062 PMC9190054

[40] Krischer JP , Gopal-Srivastava R , Groft SC , et al. The rare diseases clinical research networks organization and approach to observational research and health outcomes research. J Gen Intern Med. 2014;29(Suppl 3):S739–744. doi: 10.1007/s11606-014-2894-x. 25029976 PMC4124127

[41] Burr JS , Johnson AR , Vasenina V , et al. Implementing a central IRB model in a multicenter research network. Ethics Hum Res. 2019;41:23–28. doi: 10.1002/eahr.500016. PMC7236440 31108575

[42] Major Extremity Trauma Research Consortium. Building a clinical research network in trauma orthopaedics: the Major Extremity Trauma Research Consortium (METRC). J Orthop Trauma. 2016;30:353–361. doi: 10.1097/bot.0000000000000549. 27333458

[43] Burson KD , Walter MH , Auman JO , et al. Developing a cost-effective and efficient safety case management solution for data coordinating center projects. Contemp Clin Trials. 2025;150:107809. doi: 10.1016/j.cct.2025.107809. 39826828

[44] Zhang Y , Lin Y-Y , Lal LS , et al. Stakeholder-driven multi-stage adaptive real-world theme-oriented (SMART) telehealth evaluation framework: a scoping review. Lancet Reg Health Am. 2025:44:101041. doi: 10.1016/j.lana.2025.101041. 40115600 PMC11925543

[45] Freel SA , Snyder DC , Bastarache K , et al. Now is the time to fix the clinical research workforce crisis. Clin Trials. 2023;20:457–462. doi: 10.1177/17407745231177885. 37264897 PMC10504806

[46] Cagnazzo C , Testoni S , Speranza D , et al. The role and challenges of clinical research coordinators: insights from a national survey. Bmc Med Res Methodol. 2025;25:238. doi: 10.1186/s12874-025-02687-y. 41131448 PMC12551268

[47] Knapke JM , Jenkerson M , Tsao P , et al. Academic medical center clinical research professional workforce: part 2 - issues in staff onboarding and professional development. J Clin Transl Sci. 2022;6:e81. doi: 10.1017/cts.2022.412. 35949655 PMC9305080

[48] Palmer L , Morones SE , Jacobe HT , et al. Completion of a standardizable competency-based research training program improves understanding and preparedness for both new and experienced clinical research professionals. J Clin Transl Sci. 2025;9:e19. doi: 10.1017/cts.2024.690. 39911934 PMC11795864

[49] Rojewski JW , Choi I , Hill JR , et al. Career orientation and perceived professional competence among clinical research coordinators. J Clin Transl Sci. 2019;3:234–244. doi: 10.1017/cts.2019.385. 31660248 PMC6813517

[50] Samuels E , Champagne E , Lyden AK , et al. Implementing a mentoring program for clinical research professionals: a novel professional development initiative for university health research staff. J Clin Transl Sci. 2023;7:e247. doi: 10.1017/cts.2023.655. 38033702 PMC10685257

[51] Kamel IS. The role of robotics and automation in surgery: critical review of current and emerging technologies. Futurity Med. 2023;2:23–35.

[52] Unadkat N , Gupta S , Vinod V , et al. Robotics and automation in healthcare. In: Gupta S , Chaudhary A , eds. Artificial Intelligence in Healthcare. Boca Raton, FL: Apple Academic Press, 2026: 287–318.

[53] University data Coordinating Centers (UNICORN). University data coordinating centers, secondary university data coordinating centers, https://unicorn-dcc.org/) Accessed April 19, 2026.

[54] Boyle RM , Fortner CA , Chinchilli VM , et al. Data coordination and management in the growth failure in children with renal diseases study. J Pediatr. 1990;116:S28–S31. doi: 10.1016/s0022-3476(05)82921-x. 2405132

[55] Overton HH. Perceptions of the coordinating center: as viewed by a clinic coordinator. Control Clin Trials 1980;1:133–136. doi: 10.1016/0197-2456(80)90017-3. 7261608

