# Supplementary material for: Evolving roles of Data Coordinating Centers in multisite research: Challenges and adaptations from a rapid scoping review
Source: J Clin Transl Sci. 2026 May 15;10(1):e92. doi: 10.1017/cts.2026.10755 (PMC13237188; doi:10.1017/cts.2026.10755)
Supplement: Zhang et al. supplementary material [file S2059866126107559sup001.docx]

**Supplementary Material**

**Search Strategy**

PubMed

Search conducted on September 20, 2025

| **Search** | **Query** | **Records retrieved** |
| --- | --- | --- |
| #1 | ("data coordinating center*"[tiab] OR "data coordination center*"[tiab] OR "statistical coordinating center*"[tiab] OR "coordinating center*"[tiab] OR "data management center*"[tiab] OR "clinical coordinating center*"[tiab] OR "biostatistics center*"[tiab] OR "biostatistical center*"[tiab] OR "statistical center*"[tiab] OR "biostatistics core*"[tiab]) | 2,019 |
| #2 | ("Multicenter Studies as Topic"[Mesh] OR "multicenter"[tiab] OR "multisite"[tiab] OR "multi-site"[tiab] OR "multi-center"[tiab] OR "multi-centre"[tiab] OR "multicentre"[tiab] OR "research consortium*"[tiab] OR "clinical trial network*"[tiab] OR "research network*"[tiab] OR "study network*"[tiab] OR "collaborative stud*"[tiab] OR "multi-institutional"[tiab] OR "cooperative group*"[tiab] OR "coordinated stud*"[tiab]) | 333,711 |
| #3 | ("Quality Assurance, Health Care"[Mesh] OR "Data Management"[Mesh] OR "Information Technology"[Mesh] OR "Organizational Innovation"[Mesh] OR "Computer Systems"[Mesh] OR "Program Evaluation"[Mesh] OR "challenge*"[tiab] OR "barrier*"[tiab] OR "problem*"[tiab] OR "lesson* learned"[tiab] OR "best practice*"[tiab] OR "governance"[tiab] OR "compliance"[tiab] OR "collaboration"[tiab] OR "data quality"[tiab] OR "workforce"[tiab] OR "emerging technolog*"[tiab] OR "innovation"[tiab] OR "SWOT"[tiab] OR "SWOT analysis"[tiab]) | 3,859,078 |
| #4 | 1 AND 2 AND 3 | 185 |
| Limited to publications in English between January 1, 2010, and August 31, 2025. | | 107 |

Scopus (Elsevier)

Search conducted on September 20, 2025

| **Search** | **Query** | **Records retrieved** |
| --- | --- | --- |
| #1 | TITLE-ABS-KEY("data coordinating center*" OR "data coordination center*" OR "statistical coordinating center*" OR "coordinating center*" OR "data management center*" OR "clinical coordinating center*" OR "biostatistics center*" OR "biostatistical center*" OR "statistical center*" OR "biostatistics core*") | 3,033 |
| #2 | TITLE-ABS-KEY("multicenter" OR "multi-site" OR "multisite" OR "multi-center" OR "multi-centre" OR "multicentre" OR "research consortium*" OR "clinical trial network*" OR "research network*" OR "study network*" OR "collaborative stud*" OR "multi-institutional" OR "cooperative group*" OR "coordinated stud*") | 605,334 |
| #3 | TITLE-ABS-KEY("challenge*" OR "barrier*" OR "lesson* learned" OR "best practice*" OR "governance" OR "compliance" OR "collaboration" OR "data quality" OR "workforce" OR "emerging technolog*" OR "innovation" OR "technological innovation" OR "capacity building" OR "SWOT" OR "SWOT analysis") | 6,913,780 |
| #4 | 1 AND 2 AND 3 | 197 |
| Limited to publications in English between January 1, 2010, and August 31, 2025. | | 143 |

Web of Science Core Collection (Clarivate Analytics)

Search conducted on September 20, 2025

| **Search** | **Query** | **Records retrieved** |
| --- | --- | --- |
| #1 | TS=("data coordinating center*" OR "data coordination center*" OR "statistical coordinating center*" OR "coordinating center*" OR "data management center*" OR "clinical coordinating center*" OR "biostatistics center*" OR "biostatistical center*" OR "statistical center*" OR "biostatistics core*") | 2,113 |
| #2 | TS=("multicenter" OR "multi-site" OR "multisite" OR "multi-center" OR "multi-centre" OR "multicentre" OR "research consortium*" OR "clinical trial network*" OR "research network*" OR "study network*" OR "collaborative stud*" OR "multi-institutional" OR "cooperative group*" OR "coordinated stud*") | 489,272 |
| #3 | TS=("challenge*" OR "barrier*" OR "lesson* learned" OR "best practice*" OR "governance" OR "compliance" OR "collaboration" OR "data quality" OR "workforce" OR "emerging technolog*" OR "innovation" OR "technological innovation" OR "capacity building" OR "SWOT" OR "SWOT analysis"） | 5,206,277 |
| #4 | 1 AND 2 AND 3 | 117 |
| Limited to publications in English between January 1, 2010, and August 31, 2025. | | 89 |
